# Optimizing donor scheduling before recruitment: An effective approach to increasing apheresis platelet collections

**DOI:** 10.1371/journal.pone.0198062

**Published:** 2018-05-30

**Authors:** Parvez M. Lokhandwala, Hiroko Shike, Ming Wang, Ronald E. Domen, Melissa R. George

**Affiliations:** 1 Department of Pathology, Pennsylvania State M. S. Hershey Medical Center and College of Medicine, Hershey, Pennsylvania, United States of America; 2 Department of Pathology, Johns Hopkins University School of Medicine, Baltimore, Maryland, United States of America; 3 Department of Public Health Sciences, Pennsylvania State College of Medicine, Hershey, Pennsylvania, United States of America; European Institute of Oncology, ITALY

## Abstract

**Background/Aims:**

Typical approach for increasing apheresis platelet collections is to recruit new donors. Here, we investigated the effectiveness of an alternative strategy: optimizing donor scheduling, prior to recruitment, at a hospital-based blood donor center.

**Methods:**

Analysis of collections, during the 89 consecutive months since opening of donor center, was performed. Linear regression and segmented time-series analyses were performed to calculate growth rates of collections and to test for statistical differences, respectively.

**Results:**

Pre-intervention donor scheduling capacity was 39/month. In the absence of active donor recruitment, during the first 29 months, the number of collections rose gradually to 24/month (growth-rate of 0.70/month). However, between month-30 and -55, collections exhibited a plateau at 25.6 ± 3.0 (growth-rate of -0.09/month) (p<0.0001). This plateau-phase coincided with donor schedule approaching saturation (65.6 ± 7.6% schedule booked). Scheduling capacity was increased by following two interventions: adding an apheresis instrument (month-56) and adding two more collection days/week (month-72). Consequently, the scheduling capacity increased to 130/month. Post-interventions, apheresis platelet collections between month-56 and -81 exhibited a spontaneous renewed growth at a rate of 0.62/month (p<0.0001), in absence of active donor recruitment. Active donor recruitment in month-82 and -86, when the donor schedule had been optimized to accommodate further growth, resulted in a dramatic but transient surge in collections.

**Conclusion:**

Apheresis platelet collections plateau at nearly 2/3^rd^ of the scheduling capacity. Optimizing the scheduling capacity prior to active donor recruitment is an effective strategy to increase platelet collections at a hospital-based donor center.

## Introduction

Platelets play a major role in primary hemostasis and are transfused to patients for a number of reasons, primarily to stop or prevent life-threatening bleeding.[1] Although an effective and commonly used blood-product, platelets are one of the most challenging blood-products to manage in the blood-bank inventory. Platelets have a short shelf-life of five days after collection, of which approximately two days are spent in quarantine awaiting results of infectious disease testing.[2] Platelets may be collected either by apheresis or by pooling separated blood products from whole-blood collections. Pooling separated blood products requires platelets from five to six individual whole-blood collections to make a single adult dose (typically defined as > 3x10^11^ platelets). Hospital-based donor centers do not always have sufficient whole-blood donors to pool platelets from the same ABO-groups to create a full adult-dose of platelets. These insufficient doses may be used for pediatric patients, but are often not utilized.

Increasingly, platelets are collected by apheresis rather than whole blood collections.[3] Apheresis collections are an efficient way of obtaining platelets because an apheresis donor may donate an equivalent of up to three adult doses of platelets during each donation. Therefore, apheresis collections may yield as much as 18 times the amount of platelets compared to one whole-blood collection.[4] However, several challenges are associated with apheresis collections, which include—(a) the significant costs associated with apheresis collection machines and kits, (b) additional training of required collection personnel, and (c) a longer time commitment from apheresis donors, ranging from 1 to 2.5 hours depending on the amount of platelets that may be safely collected from one collection. Even though apheresis technology has been available for many years, relatively few donors are willing to donate via apheresis due to time constraints or lack of familiarity with the technology. Additionally, not every donor has good peripheral veins to support a long apheresis procedure. Therefore, hospital-based donor centers often are unable to meet the demands of their patients and rely heavily on regional donor centers for maintaining their platelet inventory.

Platelets obtained from regional blood centers are expensive, which is a major financial burden.[5] Additionally, during inclement weather, when the supply from the regional blood centers is disrupted even for a few days, the platelet inventory in a hospital-based blood bank can reach critically low levels. Therefore, in the interest of patient care, as well as from a financial perspective, hospital-based donor centers are incentivized to collect as many platelet units “in-house” as possible.

The typical reflexive approach to increasing platelet collections at a hospital-based donor center is to actively recruit more apheresis platelet donors. In this report, we document the effectiveness of an alternative approach: optimizing the apheresis donor scheduling capacity prior to donor recruitment efforts. We show that even with a modest number of apheresis donors at a 615-bed tertiary care academic hospital, we were able to significantly increase platelet collections by optimizing the apheresis donor scheduling capacity, in absence of active donor recruitment. When donor recruitment efforts are timed with optimized scheduling capacity, it results in a dramatic increase in apheresis platelet collections. We provide a summary of practical considerations associated with the strategy and a flowchart to guide decision between donor recruitment and expanding scheduling capacity to maximize platelet collections at hospital-based donor centers.

## Materials and methods

The study was approved by the Institution Review Board of Pennsylvania State University College of Medicine (Study 990), Hershey, Pennslvania USA. Informed consent was not required as only non-identifying data were collected and analyzed.

### Apheresis donor information and database

An analysis of apheresis collections at the hospital-based donor center was performed from November 2007 (the first day of apheresis collection at our institution) through March 2015 (89-months). Local and regional apheresis platelet donor information was obtained from the Blood Bank Control System (BBCS) database, maintained by the regional blood bank.

### Platelet collections

Platelets were collected using the Trima Accel® Automated Blood Collection System and tubing sets (Terumo BCT), per manufacturer’s protocols.

### Statistical analysis

The 89-month period was separated into four phases: growth-phase 1, plateau-phase, growth-phase 2 and donor-recruitment phase. We calculated the growth rate (slope) of apheresis collections during each phase by performing a simple linear regression model. We utilized segmented time-series analysis to perform a pair-wise comparison of the growth-trends among the first three phases to test if they were statistically different. The assumption of autocorrelation was investigated using a generalized Durbin-Watson test and a correction was performed if autocorrelation was detected. Autocorrelation correction was necessary when comparing growth-phase 1 with plateau-phase, and comparing growth-phase 1 with growth-phase 2; correction was not required in the comparison between the plateau-phase and growth-phase 2. A p-value of < 0.05 was considered significant for all statistical tests. All statistical analyses were performed using SAS software, version 9.4.

## Results

### Apheresis collections plateau at 2/3^rd^ of scheduling capacity

**Fig 1:**
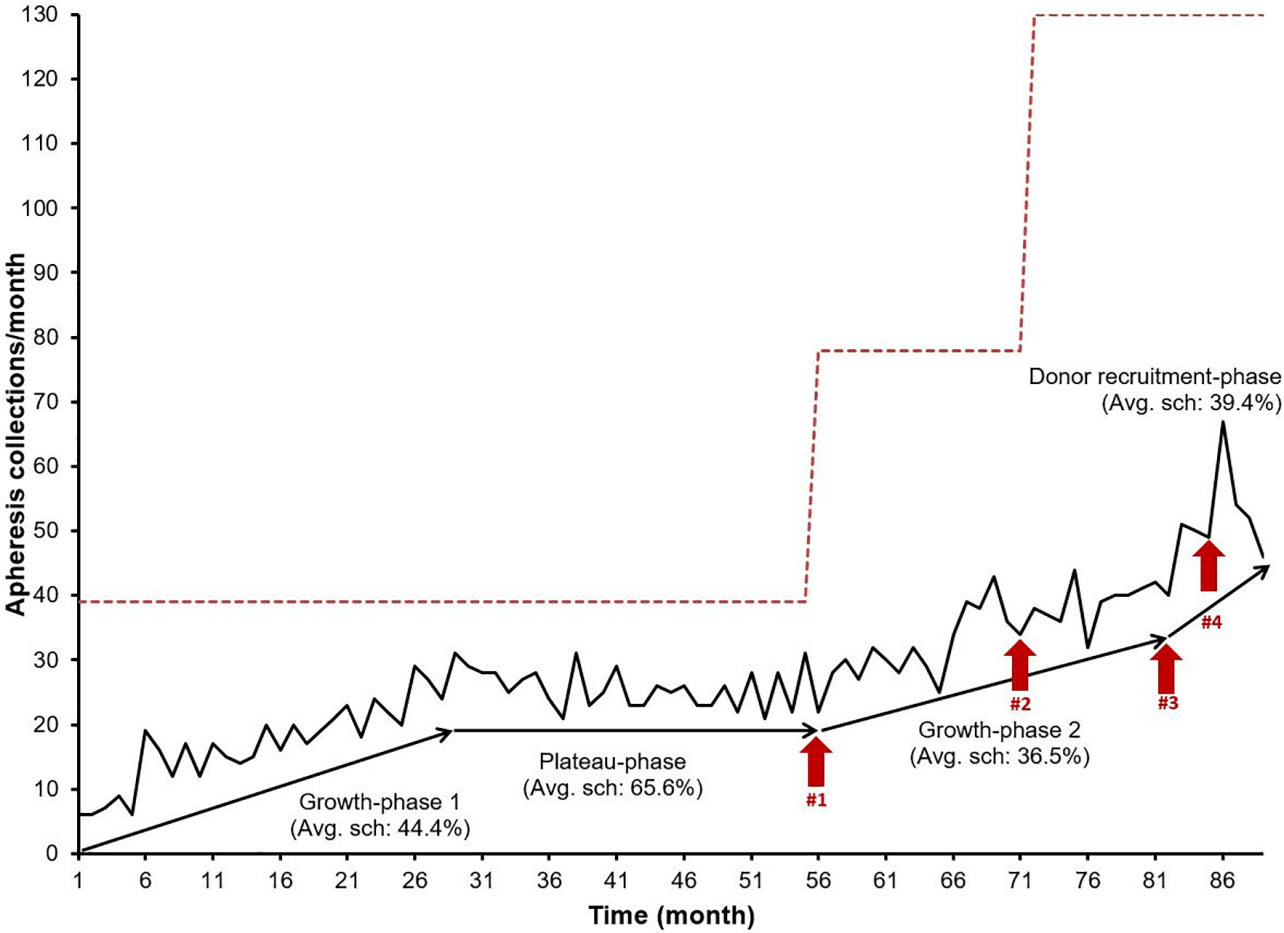
Apheresis platelet collections at a hospital-based donor center. Graph shows an 89-month analysis of apheresis platelet collections separated into four phases. (a) Growth-phase 1: Initial growth-phase prior to saturation; (b) Plateau-phase: period of no growth coinciding with schedule saturation; (c) Growth-phase 2: period of growth in collections secondary to interventions: #1—acquiring a second apheresis unit and #2—increasing donor collection days; (d) Donor recruitment-phase: transient surge in platelet collections secondary to interventions #3—scripted phone calls and #4—posting of donor recruitment flyers. The dotted line denotes the maximum scheduling capacity. Avg. sch. = Average Scheduling.

We performed an analysis of all apheresis platelet collections between November 2007 (month-1) and March 2015 (month-89) at our institution (Fig 1, Table 1). Between month-1 and month-55, we had one operational apheresis machine, which allowed the facility to operate at an average scheduling capacity of 39 collections per month (schedule of three times per day, three days per week). From month-1 to month-29, apheresis collections steadily increased from 6/month to 31/month, with linear regression showing a growth rate of 0.70 collections/month (R^2^ of 0.80). However, between month-30 and month-55, apheresis collections stopped growing and averaged approximately 25.58 collections per month (linear regression showing a growth rate of -0.09 collections/month, R^2^ of 0.05). Time-analysis showed that the growth rate during plateau-phase was statistically different when compared to growth-phase 1 (p value < 0.0001). This “plateau” in our apheresis collections may be explained by one of two possibilities: insufficient number of active donors or insufficient donor scheduling capacity (i.e. schedule filled approaching saturation).

**Table 1:** 

Month	Phase	Average schedule filled (%)	Average collections/month	Growth rate
Month-1 to -29	Growth-phase 1	44.4 ±17.1	17.3 ± 6.7	0.70
Month-30 to -55	Plateau-phase	65.6 ±7.6	25.6 ± 3.0	-0.09
Month-56 to -81	Growth-phase 2	36.5 ±7.8	34.5 ± 5.9	0.62
Month-82 to -89	Donor recruitment-phase	39.3 ± 5.9	51.1 ± 7.7	0.92

During the plateau-phase, which lasted 26 months, we performed 665 apheresis procedures at our center from 80 platelet donors, an average of 3.84 collections/donor/year. The FDA regulation allows for a maximum of 24 collections/donor/year. In comparison, at a regional blood center, platelet donors donated an average of 8.64 collections/donor/year between August 2013 and July 2014. Therefore, donors at our hospital-based donor center were donating less frequently than the regional donor center, presumably due to restrictive scheduling. This dissimilarity may also be explained by potential differences in donor demographics or more aggressive donor recruitment by the regional donor center.

A retrospective look at the percentage of the apheresis schedule capacity filled by our donors (hereafter referred to as *% schedule filled*) identifies the likely reason for the plateau. During growth-phase 1, the *% schedule filled* increased from 15% to 80% (average of 44.4%). However, during the plateau-phase the *% schedule filled* averaged 65.6% (within a narrow range from 54% - 80%) (Fig 1). Therefore, we suspect that the donor schedule was approaching saturation and was likely responsible for the lack of growth in apheresis collections during the plateau-phase.

### Increasing donor scheduling capacity renewed growth of apheresis platelet collections

We increased our total donor scheduling capacity by the following two interventions. The overall effects of these interventions are summarized in Fig 1 and Table 1.

#### Intervention 1: Obtained an additional apheresis machine

We obtained a second apheresis machine, which became operational during month-56. This intervention doubled our maximum scheduling capacity from 39/month to 78/month and consequently decreased the *% schedule filled* (Fig 1).

#### Intervention 2: Increased collection days

Since month-68, we accommodated two of our donors, per their request, on days other than the three regular scheduling days. We formally increased our operation from 3 days/week to 5 days/week from month-72 onward, further increasing our total scheduling capacity to nearly 130/month.

Collectively these interventions reduced *% schedule filled* from an average of 65.6% during the plateau-phase to an average of 36.5% (range of 28 to 55%) during growth-phase 2. Concurrently, our monthly apheresis collections during growth-phase 2 increased from an average of 25.58 collections/month during the plateau-phase to 42.0 collections on month-81, at a growth rate of 0.62 collections/month (R^2^ value of 0.64), in absence of active donor recruitment. The growth rate during growth-phase 2 was significantly different than the plateau-phase (p <0.0001), but not significantly different when compared to growth-phase 1 (p = 0.43).

### Increasing donor scheduling capacity resulted in financial savings

We acquired the second apheresis machine on a reagent-rental contract, wherein there was no upfront capital expenditure. However, the cost of the apheresis machine was captured in the adjusted price of the apheresis kits. During the 26 months of growth-phase 2, our apheresis collections averaged 34.46/month, which was an increase from an average of 25.58/month during the plateau-phase. An additional 230.88 ((34.46–25.58)/month x 26 months) apheresis platelet collections were performed during growth-phase 2. This equates to 344 adult doses, based on our split-rate of 1.49 doses/collection. The direct costs associated with in-house platelet collection was $297.68/dose, which included the technologist’s time, the cost of apheresis kits, cost of infectious disease testing, maintenance cost of the second apheresis machine, marketing cost and the cost associated with the usage of the electronic system. Indirect costs associated with platelet collections were not included in our analysis, as they were largely unchanged from plateau-phase to growth phase-2. Each dose of platelet collected in-house yielded a savings of approximately $212.32 to our institution when compared to purchasing those units from the regional blood center ($510.00/dose at the time). Therefore, our institution saved approximately $73,038.08 in direct costs during the growth-phase 2 ($212.32 x 344 doses).

### Predicting the next plateau in apheresis collections

We attempt to predict the next plateau in our collections. Our new schedule capacity was 130 collections per month. Previously, we reached a plateau when our *% schedule filled* reached 65.6%. Because our collection practices have not changed significantly, we therefore predict that our new schedule will also reach a plateau when nearly two-thirds of the schedule has been filled (i.e. 85.3 collections per month), given adequate number of donors. However, we would exhaust our apheresis platelet donors before achieving this new theoretical plateau. At the end of growth-phase 2, we had 72 active donors. Assuming the donation behavior of regional donors who donated 8.64 times/year, we expect to exhaust our current pool of donors at approximately 56.16 apheresis collections/month.

### Donor recruitment-phase

Post-interventions, our total scheduling capacity increased significantly. We argued that we might have the potential to accommodate rapid growth of apheresis collections. We tested the effect of actively recruiting apheresis donors at this stage. We solicited platelet donation among eligible blood donors using scripted phone calls (intervention #3) on month-82 and posting donor recruitment flyers around the hospital (intervention #4) on month-86 respectively. Immediately following each recruitment strategy, we saw a surge in platelet collections: 21.9% increase in the month after intervention #3 (50 collections) and a cumulative 63.4% increment (67 collections) in the month following intervention #4, compared to the average collections of the last three months in growth-phase 2 (41 collections/month). However, the effect of our temporary phone calls and the advertisement flyers were short-lived, as the number of apheresis collections gradually drifted back to the pre-recruitment growth rate. The surge in collections indicate that a donor schedule with optimal scheduling capacity has the potential for significant growth when paired with appropriate and timely recruitment strategies.

## Discussion

In this study, we document the utility of optimizing the apheresis scheduling capacity on platelet collections, prior to donor recruitment, at a hospital-based donor center. There are several limitations of this study. Donors who cancelled (or did not show up) after scheduling are not captured in our study. The effect of interventions during growth-phase 2 are overlapping, which limits our ability to evaluate the impact of each intervention separately. Several confounding factors might affect our analysis, including seasonal variations in donations, changes in donor demographics over time, increased awareness of apheresis donations in the community, etc. Because our individual study periods (growth-phase 1, plateau-phase and growth-phase 2) are each multi-year long, we predict that the effect of any seasonal or transient confounding factor would likely be minimal, if any.

### What is optimum scheduling capacity?

Hospital-based donor centers have limited resources to dedicate towards donor recruitment and management. Few studies till date have focused on computer simulations for blood donors scheduling.[6–8] Pratt et. al. used computer simulation model to study work flow and queuing problems in blood collections.[6] Bosnes et. al. used statistical models to predict blood donor arrivals in fixed appointments, whereas Testik et. al. simulated donor arrival without fixed appointments.[7,8] Such studies might be useful with staffing of donor centers. To our knowledge, no study has addressed the adequacy of scheduling capacity at a fixed-site donor center.

In this report, we provide the means to identify the lack of growth in apheresis collections which may be due to either donor scheduling approaching saturation or due to the lack of apheresis donors. Surprisingly, with a modest platelet donor database at our institution of <70 active donors (defined as donors who donated in the previous 12 months) at the time, collections reached a plateau likely due to lack of donor scheduling capacity before exhausting our donor pool. This might be true at other institutions as well. We reached a plateau when our *% schedule filled* reached an average of 65.6% (nearly 2/3^rd^) of total scheduling capacity. Although the exact *% schedule filled* that no longer supports growth will vary by institution, we postulate that the pattern of schedule saturation will likely be similar, i.e. decreased collections/donor/year compared to other regional centers. Ability to schedule donors at their convenience is expected to be critical in collection growth. Donor scheduling capacity can be increased in one of two ways: either by obtaining an additional apheresis machine or by increasing donor hours. Both methods have their advantages and disadvantages. Purchasing an additional apheresis machine allows a donor center to instantly increase their scheduling capacity by as much as two-fold. This approach is useful for donor centers that are unable to expand their current donor hours any further, or that have several donors who prefer the same time slot. The disadvantage is that buying an additional machine might require upfront capital commitment. The advantage of the second approach of increasing donor hours is that the donor center can cater their hours according to donor preferences.

Although, we experienced financial savings at our institution, there are certain limitations with our analysis. We have not included indirect costs in our analysis as they were largely unchanged by the interventions. At other institutions, however, the indirect costs such as donor recruitment, donor retention, administration costs, rent, depreciation, etc. may significantly affect the financial analysis. The cost of training additional technologists is also not included in our analysis. The financial considerations for making the decision will likely vary by institution. Our analysis also does not capture the impact of our interventions on the ability to fulfil patient’s demands, or the freshness of platelets given to the patients.

**Fig 2:**
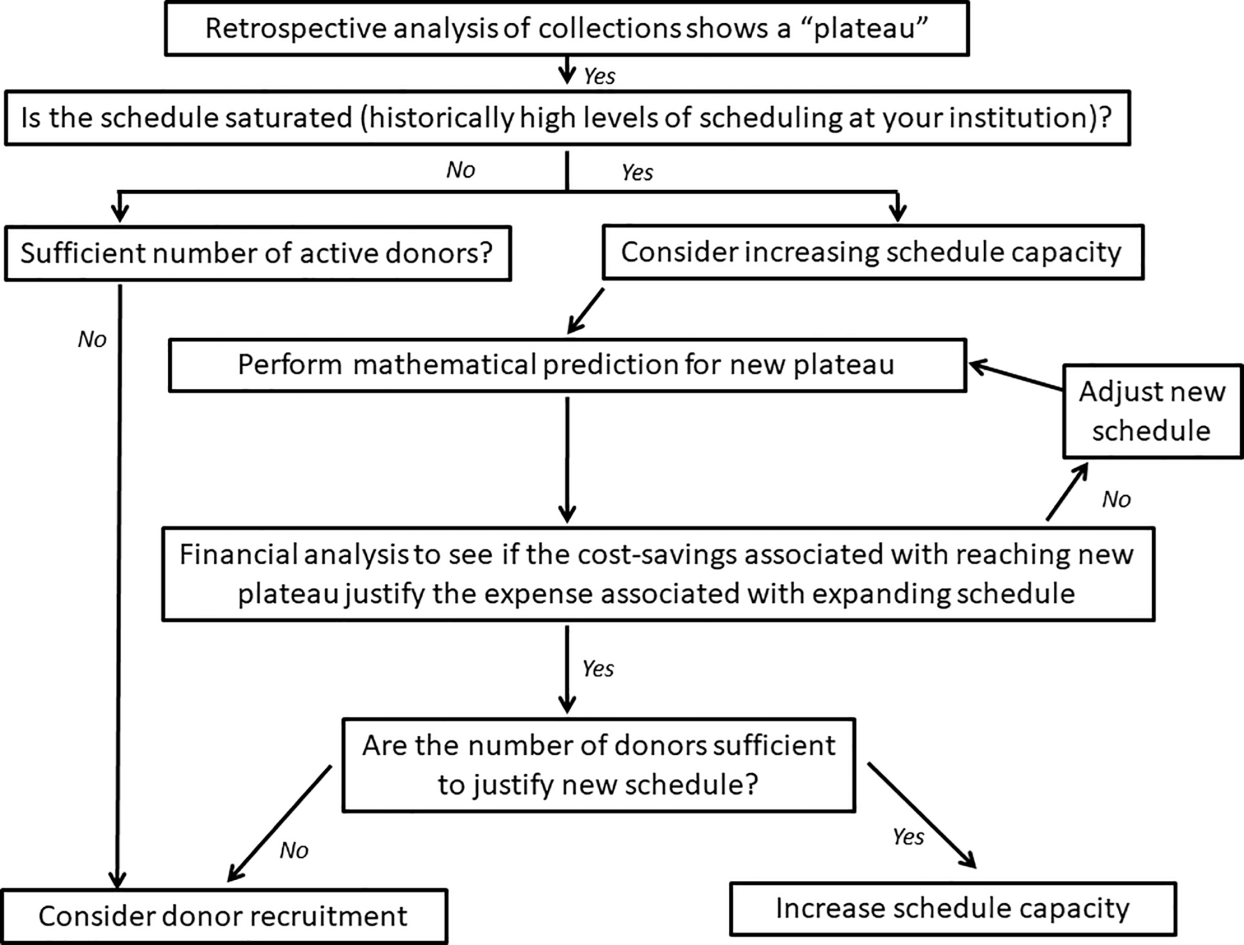
Flowchart to guide decision for increasing scheduling capacity.

We predict that if a donor center is approaching schedule saturation, then either of the above two approaches will provide a significant increase in apheresis collections. Fig 2 shows a flowchart that may be followed by institutions and managers to guide the decision between donor recruitment and expanding donor scheduling capacity in an attempt to effectively increase apheresis platelet collections.

We advise caution about generalizing interpretations from such a project. First, the operation of any individual donor center could be significantly different; therefore, the recommendation of increasing scheduling capacity is not meant as a panacea. The flowchart should be used in the context of the individual donor center’s practice. Second, our predictions are based on the assumption that the donor behavior at our institution reflects the regional average. However, the demographics of the donors at a particular donor center could be different than the regional average. Moreover, each institution and donor center has its own challenges. The prediction, while simplistic, cannot recapitulate all the possibilities or hurdles that will be encountered at any institution. For example, some hospital-based donor centers might have partnered with their regional blood centers for donor recruitment. Also, seasonal variations could be significant and dependent on the region. Finally, before expanding donor schedules, institutions should consider additional factors, particularly whether donors are available and willing to donate on the proposed new schedule. A donor survey might be useful in the planning process before implementing any changes.

## Supporting information

S1 File (XLSX)
